# Physical Education

**Published:** 1895-11

**Authors:** Harriet M. Lewis


THE
AMERICAN JOURNAL
—OF—
DENTAL SCIENCE.

VOL. XXIX. BALTIMORE, NOVEMBER, 1895. NO. 7.

ARTICLE I.

### PHYSICAL EDUCATION.

HARRIET M. LEWIS, M. D.

Our earliest life has thus been described. "The human begins life as a single cell. He passes through stage after stage of animal life owning a far away relationship to the simple creatures he so far out-strips. For a time he looks absurdly like the embryo of a fish. Later with his undeveloped heart and budding extremeties he more resembles the young bird than any other animal. On he sweeps to the mammalian form and here he can not be distinguished from an embryo pig or dog. Then his cells work away in a distinctly human direction and he becomes indistinguishable from an ape. Even here the cells never make a mistake, never grow confused and finish him into an ape but keep steadily at work until he is built into a human being."

We are beginning to see the first requisite to succeed in life is to be a good animal. The best brain is found to be of little service if there be not vital energy enough to work it.

Muscle fibre is doing the world's work. It carries our bodies to and fio. See the part it plays in respiration and the processes of digestion and excretion. In the heart and blood vessels it propels the life giving current to every organ, tissue and cell. Consider its importance in controlling brain circulation and so controlling all mental processes which result in intellectual and emotional activities. And muscle fibre brings every human being into the world.

In performing its function a muscle contracts, relaxes, rests and feeds, over and over, all day long and with the involuntary muscles, all night long, too. When a muscle contracts, the blood can not flow into it. When it relaxes, the blood flows in and in the little period of rest which follows, nutrition goes on. Force continuously applied wears out, put in the little periods of rest and labor strengthens. Watch the rythical movements of a group of men drilling. It is not exhausting work. Until we learn to relax like the cat and the dog and the baby our muscles will never be as well nourished. The woman whose muscles are starved is the woman who never stops, never relaxes and doesn't know how. We see her everywhere, in the car for instance, with rigid back wearing out sitting still. Even in sleep she does not relax but clutches the bed instead of letting it hold her. The great principle to be remembered about muscles is the principle of antagonism. Every muscle has its antagonist and the nice balancing of these is poise, health. To balance a pole, to walk a tight-rope requires very little force but what perfect muscular control, poise. The end of living seems to be growth toward perfection. What speeds a vessel on its course? Not the winds directly from behind. It is the side winds, which seems as much against as for, which fill all the sails. And so the body and mind grow strong by the balancing of antagonistic forces. The child's first utterance when he comes into the world foreshadows his destiny-struggle. One of the first things he does is to stretch out his limbs.

As the mother instinctively grasps them, he makes resistance, the first manifestation of will. Without conscious effort there is no growth of body or mind. The action and reaction between brain and muscle, between mind and body show the closest relationship. Rabbi Ben Ezra says, 'Nor soul helps flesh more now than flesh helps soul."

In a recent medical publication we read "what is the result of our highly differentiated conventional life with its pure mental ideal? The army records show a lowered physical standard, the neurologist and alienist report a marked increase of mental and nervous diseases, the general practitioner finds a large percentage of his patients needing only less mental and more muscular exercise, the teacher is forced to balance the strain of continual mental application by stimulating physical activity and the true educator recognizes that the brain is not completely trained until the higher motor centres are able to definitely and accurately control the muscles in their infinitely complicated combination and so he sees only half-trained students graduated from the various educational institutions."

Again, "It is possible for any generation to rehabilitate itself by entering fully into its physical inheritance, even so far as to overcome the effects of actual hereditary disease or of physical depression tending to the acquirement of disease."

How much irritability, bad temper, petulance, pugnacity, lack of self-confidence, which make us a pest in a home or a community are all due to physical conditions which a vigorous circulation would remove. Morbid and even feeble conditions of the body tend to generate delusions, selfishness and susceptibility to the worst impulses. Criminals as a class are undersized, undervitalized creatures, often with a deficiency of co-ordination between their faculties, sometimes with little control over their own action and no adaptability to social and industrial functions. The insane are neither strong or long lived and autopsies show disease of every organ.

"We do not expect every evil to be worked off in a gymnasium, every vice to exude through the po.es of the skin" but to a perfectly sane man or woman physically, life itself becomes a joy, the wants of mind and body can be satisfied.

Muscle fibre of good quality is something to be desired and how shall it be developed? To improve thought think, to improve muscle use it and learn to think with your muscles. Dr. Elizabeth Blackwell says, "there is as much difference between the action of thinking and unthinking muscles as between the idle gingling of tunes on the piano by a person of quick musical ear and the grand effects produced by an accomplished musician who expressing every faculty of his soul through his instrument can carry his audience with him through the wide range of passionate sentiment.

Develop muscle fibre by every out-of-door game and sport, by every manual occupation and by the gymnasium. Hygienic habits of dress, diet, air, bathing, etc., as well as true ideals for the mind and body are all indispensable requisits of body training.

The dress should allow every organ, muscle and cell to perform its function. The freedom of the gymnasium suit is like being born again and ever after a woman must have a great respect for her vital organs and ninth rib. The college girl is setting a shining example in this matter of dress and the day of the wasp waist is about over. Wear as little clothing as warmth and propriety will sanction. To carry the weight of the body on the ball of the foot, a broad sole, spring heel or low heel is necessary. On a high heel a woman comes unsteadily down and the jar to a sensitive brain and spinal cord is considerable to say nothing of the abnormal position into which the pelvic organs are thrown. Whenever practicable wear low shoes and thin soles so as to have free use of the ankle joints and muscles of the feet. Educate the feet as hands. Poise, gait depend upon it. The average human foot is not beautiful to look at but in the little child the foot is even more beautiful than the hand. Muscles of the toes are well developed by the bicycle. The Japanese baby as he bobs and blinks and squirms on the back of some child is getting muscle education especially of his feet,

It is said the Irishman temper is due to his diet of potatoes and whiskey. His butter-milk is the only thing which saves him. Pork and potatoes form too much of the farmer's diet. Poor food and monotonous labor are regarded as factors in causing the insanity of farmers' wives who make up the largest class in our state hospitals for the insane. As to what we shall eat a normal appetite is the best guide and the appetite varies with the kind of work. Emerson says even "pie was made to eat." Mrs. Rorer who knows much about food values allows her family pie but once a month. She probably has not the New England love of pie to begin with. To distinguish between a normal and abnormal appetite, a normal appetite can be satisfied, an abnormal appetite can not be satisfied. We should be no more conscious of our stomach than we are of our hearts and livers. There should be no discomfort after eating but rather a sense of well-being, every natural function should be a pleasure not a penance. The seasons themselves furnish us the best food. In theory the vegetarians have the best of it. There is nothing more revolting to one's mind than the idea of killing for food these gentle, friendly creatures to which we become so attached, when ever we see enough of them to become acquainted. It is eating our poor relations as Colonel Hambley calls them.

Oxygen is the tissue builder and we were made to live out-of-doors. Our near neighbor, the monkey, dies of tuberculosis as soon as he is shut up in the house. The North American Indian is dying off rapidly of tuberculosis and the lack of fresh air is a large part of the cause. Where they formerly lived in well ventilated camps and slept around the open camp fire they now huddle into a closely built cabin kept suffocatingly warm by a huge cook stove. The tent was moved from place to place but the little cabin is stationary and accumulates filth rapidly. It is interesting to trace the effect of habit as shown in the inheritance of the present semi-civilized Indian. Go through the dormitory at Hampton and almost invariably each Indian will have his head tucked up in a corner of his blanket and so skillfully it is not easy to find the end, while his bare feet are exposed in even the coldest nights, the old way of sleeping with his feet to the fire. We keep our houses too warm. We should accustom ourselves to lower temperatures. Put on more clothes and take more ex ercise but don't stir up the fire. 66° is warm enough for the average person, a lower temperature may be enjoyed by a vigorous one.

Bathing is as necessary as exercise and for two reasons. After vigorour exercise an immense amount of worn out material is poured out on the skin. Unless washed off, or well rubbed off, it is reabsorbed and acts as a poison. Then bathing and vigorous rubbing after exercise prevent the too rapid cooling of the surface, keeping the blood from flowing in too great quantities to internal organs and so causing congestion. The moral effect of a turkish bath is wonderful to experience. Sun baths, air baths are tonic, life giving. Benjamin Franklin used to rise early and take an air bath for an hour while doing his writing. Then he went back to bed tor his most refreshing sleep. Invalids lying for hours on the warm sand of the sea beach feel themselves growing alive again. The layer of flannel is a disadvantage. Felix Oswald says to think of taking a sun bath or swim in flannels—he should as soon think of taking ice cream in capsules.

Deep, dreamless sleep is best. In childhood when the building up processes are going on more rapidly than the breaking down processes, most sleep is needed; at maturity, when we are holding our own, less sleep and in old age more sleep. The number of hours varies with the individual.

All true emotion strengthens. All sham emotion weakens. In one of the large city hospitals the nurses were so overcome with sympathy for a new patient, an interesting young man with a serious injury, they could not eat their supper. The head nurse quietly remarked, if they had any real sympathy for the young man they would eat their supper, to gain strength to serve him.

The influence of ideals upon the development of individuals can not be strongly enough emphasized. It is said the baby's smile is copied from the mother's face and the baby features set to the pattern of the one who bends above him. How far the round shoulders, drooping head and flat chest which characterize the average student are due to the lack of a high physical ideal rather than to muscular weakness is still to be determined.

Economy is making the most of what we have, and we are wasteful not only in household economy, but in energy of mind and body. We must stop sometimes in order to begin again with a firmer step. Much energy is wasted by uncomfortable clothes, ill-fitting shoes, over-eating, unventilated rooms. We do not realize the power we use in the ordinary moments of life. Our heads should save our heels more. Most of us have some thorn in the flesh which saps in some measure our strength. Power lost through the fatigue of deafness and eye strain is worth calculating. While enthusiasm gives birth to every beneficient action, it is not well to anticipate too warmly or be too interested in anything. If everyone would do his share of the world's work there would be enough and to spare for all and how like the great world this human body is. Part of the world is starving for want of food, part of the world is starving because over-fed, "a lamp's death, when replete with oil it chokes, a stomach's when surcharged with food it starves." A man who works his brain to excess is stealing all the time from his muscles and vice versa. A normal circulation is rather the exception, a condition of congestion or æenemia too much the rule.

Darwin in his late years lamented his atrophied brain areas and he is said to have died a martyr to science. In autobiography he declares "now for many years I can not endure to read a line of poetry and I have also lost my taste for pictures and music, my mind seems to have be come a kind of machine for grinding general laws out of large collections of facts. If I had to live my life again I would have made a rule to read some poetry and listen to some music at least once every week, for perhaps the parts of my brain now atrophied would thus have kept active through use. The loss of these tastes is a loss of happiness and may possibly be injurious to the intellect and more probably to the moral character." To the woman who does not have too much of it, nothing is more restful than her sewing and a rocking chair. Geo. Sand said the needle saved her from the insane asylum. The wise man or woman has some favorite pursuit at hand, for knitting work as it were, and while giving the best strength to one line of work, keeps at least in touch with the world in as many directions as possible.

Except in acute diseases and in those conditions of the body where the knife is required, every abnormal condition may be improved by body training. The most degenerate invalid can make a beginning towards a better state of body and mind by some of the simple Swedish movements and Delsarte posings. These are the next step after rest treatment in many nervous disorders There is no possibility of body strain in the Swedish or Delsarte work, and both will keep the body in health, but to increase power apparatus must be used. Here there is danger. Unless an individual has that even development of all the faculties called "common sense" he is likely to become shipwrecked wherever he is. Every gymnasium should be under the control and constant supervision of a medical director, and while every sport and occupation of life has its accidents and calamities, this phase of the question has been exaggerated. Body training will decrease fat, correct deformities, improve pelvic disorders and remove morbid conditions due to imperfect circulation. A woman has been described as a creature with a constipated bowel and a pain in her side, and this is true of a large class. Recreation is the end of exercise. Muscles are just as susceptible to improvement as deformity and when improved tend to stay improved till abused. We must not expect too much at once from physical training. The capacity of each person is limited and changes do not become permanent in one generation. In improving ourselves we are improving still more those who come after us. When good points are acquired they are as difficult to lose as to acquire. We often see very delicate women of good family bearing robust children. Even in free America we are not all born free and equal, we are fettered by our inheritance and limited by our environment, and while it is true that a stream of itself may not rise higher than its source, "by machinery we can send a stream a good deal higher than its source and can make it do there more of vitally essential work than could all the waters of old ocean lying at their level," and training will do as much for the mind and body.

To trace briefly the history of physical education we find that four-fifths of human history is pre historic. A constant struggle with natural forces and savage foes develop man's muscle. The energy and strength of pioneers was a recompense for their labor. Later, degeneration began from disuse. In great cities it is rare to find a third generation. To regain the lost development we must return to simpler modes of living or resort to artificial methods—hence the gymnasium.

There is little to interest us in the history of education prior to the Greeks. Greece is the fountain head of western civilization and physical education was its basis. The results aimed at by physical training were—

*First.* Courage, strength and development of weak parts.

*Second.* To furnish a basis of mental development.

*Third.* The cultivation of form and beauty.

How well they succeeded in the first let Marathon tell. What they accomplished in the second is shown by the fact that they rose in less than two hundred years from a state of barbarism to a high civilization. in the third, they have given us in their art the most perfect proportions. They were ne anatomists. Their observation was the keenest but they knew nothing of modern physiology.

Wm. T. Harris says, "after the development of the personal work of art in the shape of beautiful youth by means of the national games, and the cultivation of the taste of the entire people through the spectacle of these games, was the art of sculpture by which these forms of beauty, realized in the athlete and existing in the minds of the people as ideals, were fixed in stone. Thus Greek art was born. The perfection of form still commands the admiration of the world, yet what delights us most in greek art is not the anatomical exactness to living bodies, but their gracefulness and serenety, their classic repose, whether the Gods and heroes are represented in action, or sitting and reclining postures, there is this undwelling vital activity. In the greatest activity there is considerate purpose and perfect self-control manifested. The repose is of the soul and not a physical repose. Even sitting and reclining figures are filled with activity so that the repose is one of voluntary self-restraint and not the repose of the absence of vital energy. They are gracefulness itself." And had the Greeks also left us statues of an average youth and maiden our debt to them would be much increased. We could then compare them with Dr. Sargent's figures of the typical male and female student of twenty-one years. We could estimate the changes which have resulted from our different life. Our ideals of form are largely artificial, grotesque fashions of dress being substituted for the simple attire of the greeks. With the Greek the dress was always subordinate to the form itself and so the ideal was kept pure and high. It is said that the one thing in which a nation excels is never afterwards reached by another people. Whether this need be true or not, Greece still stands as an object lesson in physical education. In Sparta woman occupied a much higher position than in the rest of Greece, and boys and girls were trained in the same gymnasium. A foreigner said to the wife of King Leonidas, "You are the only women who can maintain an ascendancy over men." "Undoubtedly" was the reply "for we are the only women who can bring men into the world." Again, in one of the plays of Aristophanrs, an Athenian woman exclaims to a Spartan sister, "How beautiful you are, how fresh your skin, how swelling your form. You could strangle an ox." The mild climate of Greece made it possible to live almost entirely out-of-doors and very little clothing was required. Speaking of Greek dress, one feature of it, the girdle, like the Japanese obi, was utterly bad. The skin was kept in order by baths and massage, and the food of the Greek was the simplest, rather a spare diet.

Among the Romans there was no spirit of the beautiful, the idea was to train soldiers; fighters. War brought wealth. Then scholars and gentlemen gave up athletics to the professional athlete, the gladiator.

In the early centuries, as Christianity began to degenerate, physical culture seemed brutal and the body was despised. To the joy-loving Greek the mind and body were co-workers. Love of bodily perfection and love of learning went hand in hand. To the Christian the body was the source of all evil. For long, weary years Simeon Stylites, his body foul with disease, stood on his lofty pedestal and refused to be washed and clean. The over-stimulation of the emotional nature gives rise to fanaticism and moral epidemics.

Chivalry revived physical training and fencing, boxing, dancing, wrestling and horsemanship were again cultivated and continued to the fall of the feudal system.

The German system of physical education has been more like the Roman, while the English have adopted the the sports of Greece.

In sweeden, Ling, (1777-1839) patriot, gymnast and poet, was inspired with a desire to revive the ancestral spirit in the Sweedish people by the help of sport and song, to draw out once more the great qualities, the strength, courage and will, which in old times had distinguished the Scandinavian race. After a hard struggle he succeeded in 1814 in founding the Royal Central Gymnastic Institution, where all the teachers of gymnastics throughout the country receive their education Training is obligatory in every school and the fine bearing of the Sweedish children is said to be noticeable. Swedish medical gymnastics have been introduced into almost every country.

In France, Delsarte did a great work without realizing the tendency of his teachings in the line of physical education. His own daughter Madame Geraldry is said to have declared "It is the expression of the emotions through the body that my father taught. If one wishes to study gymnastics, let him go to a gymnasium." Delsarte, the son of a physician, was born in 1811. After years of labor and study, which took him by turns to hospitals, morgues, asylums, prisons, art galleries, etc., patiently unearthing the secrets and methods of past genius, study which kept him enchained by the hour watching the children at play in the great publ c gardens, weighing humanity every where and every how, he succeeded in formulating the laws of æsthetic science. He died in 1871 without arranging his life work for publication. Delsarte trained actors, and many are the famous names that have owed much to his instruction. By posture, jesture and facial expression the story is told before the lips utter a sound. This supposes a great amount of muscle control. The inspiring motive accompanying every act makes every movement a pleasure, and attracts temperaments to whom the mechanical work of a gymnasium would be utterly wearisome. Delsarte realized the necessity of preserving a just proportion between nerve force and muscle and taught the phychic transcends the physical always. Gymnastic systems measure results too much by external growth, Delsarte was called an artist philosopher and his philosophy is as fascinating as the truth. Here are a few extracts. "Art is not an imitation of nature but nature illuminated. It is the surest, purest and most constant good in life." "The true artist does not grow old. He is never too old to feel the charm of beauty. The more a soul has been deceived the more it has been chastened by suffering, the more susceptible it is to the benefits of art. This is why music soothes our sorrows and doubles our joys. Song is the treasure of the poor. On his desk after his death was found this sentiment. "Art is an act by which life lives again in that which in itself has no life."

### PHYSICAL TRAINING IN AMERICA.

Our forefathers needed no gymnasium, but now, in the crowded city especially, as life grows easier, physical and mental degeneration is progressing. Benjamin Franklin seems to have been the first enthusiastic supporter of physical training. He and Thomas Jefferson advocated abundant exercise in connection with education. At the present time the Swedish system, the German, Delsarte, Dr. Sargent's system all have warm supporters. If the work of Dr. D. A. Sargent of Harvard were expunged from the field of gymnastics it would be found that America's original contributions to the cause have been lamentably few. But with his inventions America is able to make a very respectable showing of originality. He imitates in the gymnasium the natural experiences which have developed the race. Dr. Sargent says, "What America most needs is the happy combination which the European nations are trying to effect, the strength-giving qualities of the German system, the active and energetic properties of the English sports, the grace and suppleness acquired from the French calisthenics and the beautiful poise and mechanical precision of the Swedish free movements, all regulated, systematized and adapted to our peculiar needs and institutions. The highest development of strength, activity and grace is not compatible in the same individual and consequently many persons prefer to sacrifice one in order to gain the other, but life's forces are well adjusted and well balanced."

In the public school, gymnastics as at present conducted, can accomplish little save to equalize in some measure the circulation. Putting the will into the muscles, when the will is already tired, is only making a still greater drain on nervous energy. Severe mental work cannot be compensated for by severe physical work. It is rest for the will which is needed, and the will is rested by yielding to one's humor. Every pound of energy expended on work, either of mind or of body, must be made good by food, rest or sleep. Plato's description of the Greek athletes, "stupid and lazy fellows who did nothing but eat and sleep," describes the condition of rest after severe physical exercise. Mental achievement is the basis of promotion in the school. Is it not high time that more attention should be directed to giving sound and well shaped bodies to the boys and girls who so soon will be the men and women upon whom the race depends, In our colleges for men and women, a resident physician is established as supervisor of gymnastics and recording inspector of physical development among the students. Observations are being reduced to statistical form and we are beginning to have a harvest of treatises. After special diagnosis, special exercise is prescribed for each student, the object in view the development of the race as a whole. Body trainers have been so busy with the practical side of the work that little has been done in the study of the physiological psychological effects of exercise. Harvard has recently established a laboratory for the experimental study of the physiology of exercise as Dr. Fitz says, "This is a clear acknowledgement of the high educational claims of physical training and a distinct advance in the history of physical education." To quote from Dr. Hitchcock of Amherst, "The gymnasium will do much but it is not the natural or ideal form of exercise. To get the real brawn, effective muscle, capacious lungs, a tough skin, the best of digestions and a really reliable heart, one must have more of the natural process of health from mother earth, from air, water, temperature, ozone and the actual touch of soil and grass. No animal, even with the utmost care, thrives in the house Hence the athletic sports have come in again. They have come to stay and we can have the necessary exhibitions without brutality, courseness, unfairness or even indelicacy."

It is the opinion of most educated Englishmen that the cultivation of foot-ball in the public schools has had not a little to do with the courage, address and energy, with which the graduates of Rugby, Eton and Harrow, have made their way through dangers and over difficulties in all quarters of the globe.

Gen. Walker says: In the competitive contests of our colleges something akin to patriotism and public spirit is developed with results on the whole of good. It is a good thing that the body of students should now and then be stirred to the very depths of their souls, that they should have something outside themselves to care for, that they should learn to love passionately, even if a little animosity towards rivals must mingle with their patriotic fervor, that they should at times, palpitate with hope and fear and anxiety in the view of objects which can bring to them personally neither gain nor loss. The contemplation of fine physical development cannot fail to secure a much wider cultivation of gymnastics than would take place without it. And how the college athletic games light up the life of our people. We need more of popular amusement that shall call us out into the open air in great numbers, of more that shall arouse an interest in something besides money getting and professional preferment. At a recent meeting of the Harvard chapter of the Phi Beta Kappa, the orator of the day questioned, whether college athletics have not after all a deeper significance than even the most sanguine attach to them, whether this remarkable outburst of enthusiasm for the perfecting of the human body is not related to the growth of a feeling for art in this new land of ours. It was not an accidental coincidence that the nation of the old world which pursued athletics with the most passionate eagerness was the same nation which carried the plastic arts to the highest point of perfection ever attained. The period of artistic excellence followed the highest athletic period and in the history of any nation the development of art starts late. The Life Class is the true school of the artist. The greatest of all who bear that name have been those who revered the human form, made it their chief study and found in it their highest delight. A great artist of the present day, makes this argument for the physical training of women. "All beauty is sexless in the eyes of the Artist at his work, the beauty of man, the beauty of woman, the heavenly beauty of the child which is the sweetest and best of all. Indeed, it is woman, lovely woman, whose beauty falls the shortest for sheer lack of proper physical training." To those who decry the Life Class this artist pleads a sincerity equal to theirs and as deep a love and reverence for the gracious, goodly shape that God is said to have made after his own image for inscrutable purposes of his own."

Home making is supposed to be the peculiar work for which a woman is best fitted and in which she succeeds best. No where in woman's work is there a more lamentable lack of muscle How many mothers are invalids for life after the birth of the first child because muscle fibre was not equal to the work lain upon it and never recovered from the shock of its over exertion. To make a home what it should be the average woman needs the muscle of five combined or else the still greater power, brains enough to keep five average women effectively at work. It does not matter just how a woman's muscles and nerves differ from a man's in kind or quantity, but it does matter that she have all nature meant her to have and since it is her privilege to perpetuate the race, nature never endowed her with an inferior quality of muscle fiber.

Dr. Sargent says up to thirteen years of age he will accomplish more with a class of girls than boys. The girl is more agile and at puberity is stronger and heavier than the boy. Why such a difference in strength later if not due to wrong education. After puberity Dr. Sargent would keep right on with the training of the girl but would have it different from the boys.

The king of dahomey has for his body guard a band of women, called Amazons, and they are said to be the fiercest fighters.

In those conditions of life where both sexes work out-of-doors at manual labor there is no deficiency of muscular power in the woman. Among our North American Indians the muscular power of the women is perhaps superion and her health record far better. She has gained muscular power at the expense of rounded shoulders and clumsy gait, while the man retains, in appearance at least, the fine physique which comes from out-of-door training for warfare and the hunt. Gravity, unless constantly resisted, tends to drag us down to the earth always, so we see too often in the manual occupations, as in the powerful frame of the farmer, the drooping head and bent form. The out-of door sports have an opposite effect and tend to erectness, speed and great range of movements, So we are creatures to work and to play. Compare the muscles of the cart horse with the slender limbs of the racer, both splendid specimens of power but how different in kind. The cart horse may hold his head as proudly as the racer, so the lack of an ideal may have something to do with the farmer's peculiar deformity as well as the students.

The qualities gained by body training are not only strength, swiftness and precision of touch, but steadiness of nerve, quickness of apprehension, coolness, resourcefulness, self-knowledge, self-reliance, fair play, ability to work with others, power of combination, readiness to subordinate individual impulses, selfish desires and even personal credit to a common end. These are all qualities of great usefulness in any work for man or woman and whether certain phases of physical exercise concern women directly or indirectly, the reflex influence upon her of the whole question in all its phases is a powerful one.

In the family the strongest iufluence is the physician and this work of body training, especially for the girl and woman, is his.—*Journal of Medicine and Science.*

